# Cardiovascular autonomic neuropathy in people with type 2 diabetes mellitus; investigation of its association with classical cardiovascular risk factors using cardiovascular autonomic reflex tests: a cross-sectional study

**DOI:** 10.1186/s43044-021-00168-3

**Published:** 2021-05-13

**Authors:** Ahmad Osailan

**Affiliations:** Department of Physical Therapy and Health Rehabilitation, College of Applied Medical Sciences, Prince Sattam Bin Abdulaziz University, Alkharj, Saudi Arabia

**Keywords:** Diabetes mellitus, Cardiovascular autonomic neuropathy, Cardiovascular risk factors, Autonomic nervous system, Exercise tolerance test

## Abstract

**Background:**

People with type 2 diabetes mellitus (DM) are at increased risk of cardiovascular disease (CVD). Cardiovascular autonomic neuropathy (CAN) is an underdiagnosed risk factor for CVD, which is prevalent among people with 2DM and can lead to CVD and CVD-related mortality. Little is known about the risk factors associated with CAN in type 2DM. Thus, the study was aimed to assess CAN using five cardiovascular autonomic reflex tests (represented by Ewing’s score) and explore the factors associated with CAN in people with type 2DM. The studied factors include traditional and serological CVD risk factors obtained from a fasting blood sample and cardiorespiratory fitness (CRF) obtained via attainment of the highest peak of volumes of O_2_ (VO_2Peak_).

**Results:**

Univariate analysis revealed a significant positive correlation between resting systolic blood pressure (SBP) and Ewing’s score (*r=0*.47, *p=*.02) and an inverse correlation between VO_2Peak_ and Ewing’s score (*r*=−0.64, *p*=.001). Multivariate linear regression revealed that a significant model that included resting SBP and VO_2Peak_ explained 93.8% of Ewing’s score variance.

**Conclusion:**

CAN was associated with two CVD parameters, including resting SBP and CRF, which may indicate the importance of controlling these two factors to prevent or reduce CAN in people with type 2DM.

## Background

Diabetes mellitus (DM) is a metabolic disorder characterized by an elevated level of glucose (hyperglycemia) and disturbance of fat, protein, and carbohydrate metabolism caused mainly by the inability to produce or to utilize insulin [1]. DM may start early in life and be carried for a long time asymptomatic [2]. A report showed that the number of people with DM was 366 million in 2011, and half of these people did not know they have DM [3]. DM is alarmingly growing, as it is estimated that the number of people with DM worldwide will increase up to 552 million [3].

People with DM (type 1 or type 2) have an increased risk of cardiovascular disease (CVD) [4, 5], which accounts for more than 60% of the mortality rate among people with DM [6]. The risk of death from coronary heart disease, a type of CVD, is threefold higher in people with type 2DM than non-diabetic people [7]. A cluster of CVD risk factors is prevalent in people with DM, which contribute to the increased risk of CVD, including obesity [8], hypertension [9], dyslipidemia [9], physical inactivity [10], and cardiovascular autonomic neuropathy (CAN) [11, 12].

CAN is one of the underdiagnosed DM complications and one of the major risk factors for CVD in people with DM [13]. It is known as the impairment of the nerve innervated by the autonomic nervous system (ANS) that regulates the heart and the blood vessels [14]. The prevalence of CAN is higher in type 2DM and has been reported between 31 and 73%, whereas, in type 1DM, the prevalence reported between 17 and 66% [13]. Different methodologies and tests to examine CAN have contributed to this variation in prevalence. The presence of CAN among people with DM has been correlated with an increased 5-year CVD mortality rate [15]. Symptomatic manifestations associated with the presence of CAN include resting tachycardia, postural hypotension, exercise intolerance, silent myocardial ischemia, or infarction, and left ventricular systolic and diastolic function [5, 15].

Assessment of CAN involves both arms of the autonomic nervous system (ANS), the sympathetic and parasympathetic nervous system functioning. The gold standard method to assess CAN was established by Ewing et al. [16], which include a set of five cardiovascular autonomic reflex tests. Blood pressure (BP) response to standing, Valsalva manoeuver, and BP response to sustained handgrip test assess sympathetic function, and heart rate (HR) response to standing and HR response to deep breathing are to assess parasympathetic function. These tests have shown high validity and reliability in measuring ANS function in people with DM [17].

Little is known about the risk factors associated with CAN in people with DM. In longitudinal studies, several risk factors in people with DM were linked with increased risk of CAN, including duration of the disease, glycemic control [18], and CVD risk factors (obesity, smoking, hypertension, and hyperlipidemia) [13]. Age and presence of diabetic complications such as diabetic retinopathy and nephropathy were also reported in cross-sectional studies [19–21]. However, these findings were inconclusive and not generalizable as some of these studies were limited to type 1DM [21], and the information about the CVD risk factors for CAN in type 2DM was limited. For example, low cardiorespiratory fitness (CRF) is one of the main risk factors for CVD. Yet, in cross-sectional studies, it was not included as a potential risk factor for CAN. In a comparison study between type 1DM and type 2DM, CRF was reduced in type 2DM less than type 1DM, and there was a strong association between CAN and CRF only in type 1DM [22]. Thus, due to the limited information about the risk factors associated with CAN in type 2DM, the current study was aimed to assess CAN using cardiovascular autonomic reflex tests and explore the factors associated with CAN in people with type 2DM. It was hypothesized that CAN will be associated with CVD risk factors.

## Methods

### Study population

Twenty-six people with type 2DM (HbA1C ≥ 48 mmol/mol, and fasting glucose > 7.0 mmol) were recruited from diabetes educational classes between 2015 and 2016 at Brierley Hill Health and Social Care Centre, Dudley, UK. People with comorbidity incompatible with exercise as per American College of Sports Medicine (ACSM) guidelines [23], atrial fibrillation, established CVD, established or confirmed diagnosis of CAN prior to participation, neurological problems (e.g., Parkinsonism, dementia), uncontrolled hypertension (170/80 mmHg), or uncontrolled glucose level above 200 mmol/l were excluded. Additionally, severe pulmonary disease (e.g., COPD, pulmonary fibrosis) and mental or physical impairment causing the inability to perform any test were excluded. The study was conducted according to the guidelines of the Declaration of Helsinki and approved by the National Research Ethics Committee (IRA ID: 169234, Ref: 15/EM/0138), and all participants provided written informed consent before participation.

### Protocol

**Fig. 1:**
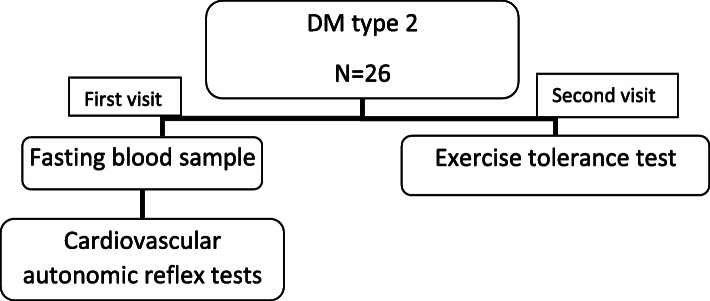
Flow chart of the assessment of the study

The protocol used in the current study is similar to what was reported by Osailan et al. [24] with the addition of cardiovascular autonomic reflex tests. Participants were invited to the research laboratory on two different occasions. During visit one, height was measured to the nearest 0.5 cm using a standard height measure (Seca 214 Road Rod), weight and body mass index (BMI) were measured using a Tanita BC 418 MA Segmental Body Composition Analyser (Tanita Corporation, Tokyo, Japan), and brachial blood pressure was taken using an electronic sphygmomanometer (Datascope Accutor, Mahwah, NJ, USA). At the same time, the participant was seated, then a fasted blood sample was taken. This was followed by performing five cardiovascular autonomic reflex tests. On the second visit, an appropriate mask was fitted to the participant covering the nose and mouth to analyze inspired and expired gases, and electrocardiograph (ECG) (12-channel ECG, Custo cardio 200, Custo med, Leipzig, Germany) electrodes were attached (see Fig. 1). Two-minute baseline measurement was used to measure resting heart rate and volume of O_2_ consumption while seated, followed by an exercise tolerance test (ETT), which included 6 min of familiarization phase. Finally, a minimum of 6 min post ETT as a recovery period.

### Collection of blood sample

Blood was collected from the patient’s antecubital vein using a butterfly needle equipped with the vacutainer system. The blood samples were analyzed for serological risk factors for cardiovascular disease, including full blood count and lipids. All analyses were carried out using routine laboratory procedures in the hospital’s research laboratory.

### Cardiovascular autonomic reflex test

Five cardiovascular autonomic reflex tests were conducted consecutively, while ECG electrodes were attached to the participant to monitor heart rate changes during specific tasks. The cardiovascular autonomic reflex tests, commonly known as Ewing’s score, are valid and reliable tests for detecting CAN [17]. The credit of the development of these tests is to Ewing. Clarke and coworkers from Edinburgh. The total scoring of Ewing’s score is based on the achieved scores from a battery of five tests, including:

- Valsalva manoeuver
- Heart rate (HR) response to standing up
- HR response to deep breathing
- Blood pressure response to standing up
- Blood pressure response to a sustained handgrip

#### Valsalva manoeuver

The participant was instructed to blow into a mouthpiece (with a closed glottis) with a pressure meter while maintaining a pressure of 40 mmHg for 15 s. The longest R-R interval ratio immediately after the manoeuver to the shortest R-R interval during the manoeuver was measured. Then, an average of three readings was taken (see Table 1 for interpretation of the test results).

**Table 1:** Cut off values for the results of the cardiovascular autonomic reflex tests

Test	Normal (zero)	Borderline (0.5)	Abnormal (1)
Valsalva maneuver	≥ 1.21	1.11–1.2	≤ 1.10
Heart rate response to standing up	≥ 1.04	1.01–1.03	≤ 1.00
Heart rate response to deep breathing	≥ 15	11–14	≤ 10
Blood pressure response to standing up	≤ 10	11–29	≥ 30
Blood pressure response to a sustained handgrip	≥ 16	11–15	≤ 10

#### HR response to standing

The participant was lying down in a supine position for 5 min, then was instructed to stand up immediately unaided without previous warning. The test provokes an increase in HR at the 15th beat, followed by a decrease in HR at the 30th beat. Thus, a ratio of 30:15 was measured and was quantified based on the longest R-R interval at the 30th beat to the shortest R-R interval at the 15th beat (see Table 1 for interpretation of the results of the test).

#### HR response to deep breathing

Six breaths per minute were elicited using a metronome for 2 min for familiarization while in a seated position. After that, every minute, the maximum HR and minimum HR were recorded for three consecutive breath cycles. The difference between the maximum and minimum HR was measured from the average readings during the three breath cycles (see Table 1 for interpretation of the test results).

#### Blood pressure response to standing

The participant was lying supine for 5 min, with an automated sphygmomanometer attached. Then at the end of the 5th min, blood pressure was measured. Then, the participant was instructed to stand up immediately, and another blood pressure reading was recorded. The difference between supine systolic blood pressure and standing systolic blood pressure was calculated (see Table 1 for interpretation of the test results).

#### Blood pressure response to a sustained handgrip

The participant was asked to practice using the dynamometer to obtain a full maximum handgrip. After 1 min of rest, resting blood pressure was measured. Then, the participant was asked to maintain 30% of the achieved maximum handgrip for 5 min. During every minute, blood pressure was measured. The difference between the resting diastolic blood pressure and the diastolic blood pressure at the end of the last minute was calculated (see Table 1 for interpretation of the test results).

### Exercise tolerance test (ETT)

Before ETT, and for the participant’s safety, it was a criterion to have capillary blood glucose between 7 and 14 mmol/L [25]. ETT was performed on a treadmill (HP Cosmos Mercury, Nussdoerf-Traunstien, Germany). Six minutes was given to each participant for familiarization purposes before commencing the individualized ramp protocol test, which was modified according to the patient’s fitness and physical abilities [26]. During this familiarization period, the speed was set at the participant’s preference (approximately three kph) with 0% inclination for 6 min. After that, the treadmill’s speed was increased up to maximum brisk walking based on the participant’s ability. Then, the speed was set to be constant (maximum brisk walking) throughout the test while increasing the inclination by 1% every minute. Peak oxygen consumption (VO_2Peak_) was calculated using breath-by-by-breath gas analyses (Metalyzer 3B, Cortex, Leipzig, Germany). Heart rate was recorded before commencement, throughout the ETT, and during the recovery period using ECG. If volitional exhaustion was reached during ETT, or any relative or absolute contraindications of ACSM’s guidelines were met [23], the test was immediately terminated. Upon the test’s termination, the participant was seated on the chair for up to 6 min as a recovery period. A cardiac physiologist supervised the tests to monitor the relative and absolute contraindications. The cardiac physiologist was blinded to the aim of the study.

### Outcome measures

#### Cardiovascular autonomic reflex test

The scoring system used for each task’s results was based on the cut-off values suggested by Ewing et al. [16]. The cut-off values are presented in Table 1. A 0 (zero) score was given for the normal result, 0.5 for a borderline result, and 1 for the abnormal result (see Table 1). Thus, each participant’s total score was between 0 and 5 out of the five tests and was presented as Ewing’s score.

#### Blood sample analysis

Blood samples were analyzed for total cholesterol, triglycerides, high-density lipoprotein (HDL), and low-density lipoprotein (LDL). Insulin resistance was analyzed using homoeostasis model assessment (HOMA) [27].

#### Cardiorespiratory fitness (VO_2peak_)

Peak aerobic capacity (VO_2peak_) was measured via calibrated breath-by-breath gas analyzer during treadmill ETT. Inspired and expired gas data were averaged every 2 s. VO_2_ reading data were further smoothened by taking the average of VO_2_ every 28 s (taking an average of 14 readings of VO_2_ ml/min). VO_2peak_ was defined as the highest VO_2_ during the test and was expressed as ml/min/kg.

### Statistical analysis

Statistical analysis was performed using SPSS27 (Chicago, IL, USA). The normality of the variables was tested using the Kolmogorov-Smirnov test. Normally distributed variables were presented as means and standard deviation. Skewed variables (body mass index, HOMA, and Ewing’s score) were presented as a median and interquartile range). Due to some of the variables’ skewness, including the main outcome measure, the bivariate spearman’s rank correlation was used to assess the relationship between Ewing’s score and other variables. Linear regression (using the enter method) was used to identify the independent factors associated with Ewing’s score. Only the variables significantly correlated with Ewing’s score in the univariate analysis were entered as independent variables and Ewing’s score as the dependent variable (all in one block). The level of significance for the analysis was set at ≤ 0.05. Power calculation analysis (GPower version 3.1) using a *priori* test indicated that the sample size required to achieve the power of (1-β error probability) = 0.85 was 26 with an effect size (*d*) of 0.55.

## Results

### Characteristics of the participants

The demographic characteristics of twenty-six type 2DM (60.8 ± 10.4 years, 38.5% female) are presented in Table 2. Comorbidities within the participants were bronchitis (3.8%), hypothyroidism (3.8%), depression (3.8%), sleep apnea (3.8%), osteoarthritis (3.8%), and osteoporosis (3.8%), and 11.5% were current smokers. The medications were not altered or discontinued at the time of the study. The most common medications used by the participants were statins (46.2%), Biguanides (53.8 %), and angiotensin-converting enzyme inhibitors (ACE inhibitors) (34.6%). Other medications are demonstrated in Table 2. All medications used by the participants were orally administered.

**Table 2:** Demographic characteristics and common CVD risk factors of the participants

Variable	*N*=26 type 2 DM
Age (years)	60.8 ± 10.4
Sex, F (%)	38.5%
Height (m)	1.7 ± .10
Weight (kg)	92.4 ± 23
BMI (kg/m^2^)	30.8 (29.1–36.7)
Body fats %	33.9 ± 6.8
Heart rate rest (bpm)	71.4 ± 12.1
Resting SBP (mmHg)	132 ± 15.7
Resting DBP (mmHg)	80 ± 9.9
Total cholesterol (mmol/L)	4.6 ± .96
Triglycerides (mmol/L)	1.4 ± .56
HDL (mmol/L)	1.2 ± .22
LDL (mmol/L)	2.8 ± .93
HOMA	2.3 (1.5–4.5)
Ewing’s score	2 (1.25–2.5)
VO_2peak_ (ml/kg/min)	22.3 ± 4.5
***Medications***	
Statins (%)	46.2%
Biguanides (%)	53.8%
ACE inhibitors (%)	34.6%
Ca channel blockers (%)	7.7%
Diuretics (%)	7.7%
Gliclazide (%)	7.7%
Fluoxetine (%)	7.7%
Alpha-blockers (%)	3.8%
Thyroxin (%)	3.8%
Rivaroxaban (%)	3.8%
Sitagliptin (%)	3.8%
NSAIDs (%)	3.8%
Beta-blockers (%)	3.8%

Values are presented as means ± standard deviation or median (25th to 75th percentile values) as appropriate

*BMI* body mass index, *SBP* systolic blood pressure, *DBP* diastolic blood pressure, *HDL* high-density lipoprotein, *LDL* low-density lipoprotein, *HOMA* homeostasis model assessment insulin resistance, *Ewing’s score* total score of the cardiovascular autonomic reflex tests, *VO*_*2peak*_ the highest volume of oxygen, *ACE* angiotensin-converting enzyme, *Ca* calcium, *NSAIDs* non-steroidal anti-inflammatory drugs

### Correlation

**Fig. 2:**
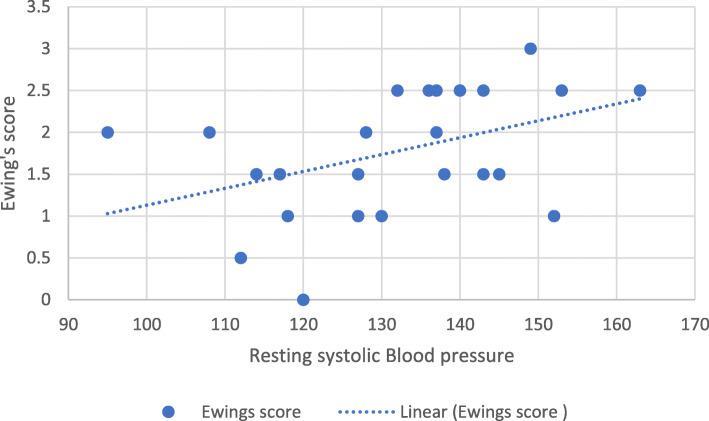
Correlation between Ewing’s score and resting systolic blood pressure

**Fig. 3:**
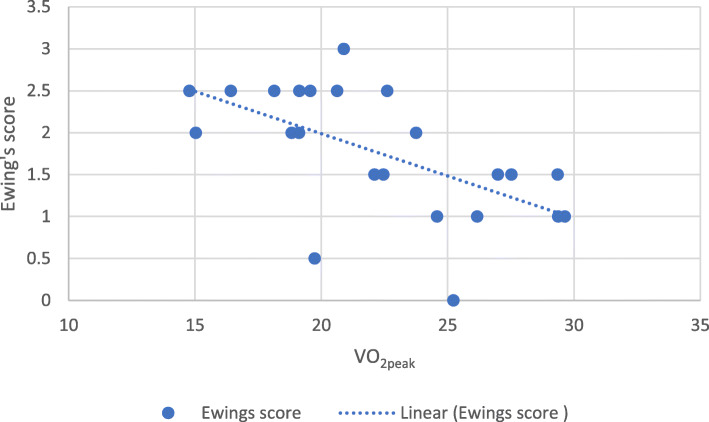
Correlation between Ewing’s score and VO_2peak_

Correlational analyses were used to assess the factors associated with Ewing’s score. Two variables were significantly associated with Ewing’s score. Resting SBP was positively associated with Ewing’s score (*r* (23) = 0.47, *p*= .02) (Fig. 2), whereas VO_2peak_ was inversely associated with Ewing’s score (*r* (21) = −0.64, *p*= .001) (Fig. 3) (see Table 3). This indicated that higher resting SBP and poor CRF are related to higher abnormal cardiovascular autonomic reflex tests.

**Table 3:** Correlation of Ewing’s score with common CVD risk factors

Variable	Ewing’s score	
	*r*	*p*
Age	0.19	.37
Sex	0.01	.98
Weight	0.24	.26
BMI	0.25	.22
Body fats	0.17	.41
Heart rate rest	0.13	.55
Resting SBP	0.47	**.02**
Resting DBP	0.09	.66
Total cholesterol	−0.12	.57
Triglycerides	0.19	.35
HDL	0.02	.93
LDL	−0.22	.31
HOMA	0.03	.89
VO_2peak_	−0.67	**.001**

*BMI*, body mass index; *SBP*, systolic blood pressure; *DBP*, diastolic blood pressure; *HDL*, high-density lipoprotein; *LDL*, low-density lipoprotein; *HOMA*, homeostasis model assessment insulin resistance; *VO*_*2peak*_, the highest volume of oxygen

*P* in bold indicates a significant association

### Linear regression

Linear regression was used to identify if related variables in the univariate analysis significantly predict Ewing’s score. Only the variables significantly correlated with Ewing’s score in the univariate analysis were entered in a multivariate linear regression analysis. Resting SBP and VO_2peak_ were entered as independent variables and Ewing’s score as the dependent variable (see Table 4). The results of the regression indicated that a significant model, which included resting SBP and VO_2peak_ explained 93.8% of the variance in Ewing’s score (*F* (2, 21) = 158.,8, *p* < .000).

**Table 4:** Linear regression model for factors associated with Ewing’s score

Variable	Ewing’s score		
	*β*	*t*	*p*
Resting SBP	1.99	8.29	<.001
VO_2peak_	−1.09	−4.51	<.001
*R*^2^ value of the model	*R*^2^		
	.938		<.001

*SBP*, systolic blood pressure; *VO*_*2peak*_, the highest volume of oxygen

## Discussion

The current study was aimed to assess the relationship between common CVD risk factors and CAN assessed by cardiovascular autonomic reflex tests (identified by Ewing’s score). Not all CVD risk factors were associated with Ewing’s score. However, only two variables were moderately associated with Ewing’s score. The study showed that Ewing’s score was positively associated with resting SBP and inversely associated with CRF (measured via VO_2peak_). Besides, the multivariate linear regression showed a significant model which included resting SBP and VO_2peak_ as independent predictors of Ewing’s score. This model explained most of the variance in Ewing’s score in the sample. This may suggest that high systolic blood pressure and low CRF contribute to worse Ewing’s score and, therefore, contribute to CAN.

According to the Ewing and colleagues method, the gold standard to measure the CAN is cardiovascular autonomic reflex tests [16]. The five tests reflect both arms of the sympathetic and parasympathetic nervous system, and a minimum of two abnormal tests define the presence of CAN in people with DM [28]. The presence of CAN with two abnormal tests was linked to higher CVD mortality rates [12]. Therefore, identifying risk factors associated with CAN may help health care professionals attenuate these factors and implement strategies to reduce future risk and mortality.

Studies investigating the risk factors associated with CAN in people with DM (type 1 and type 2) are scarce. In a study comparing between type 1 and type 2DM, a significant association was reported between the total scores of the cardiovascular autonomic reflex tests and SBP in type 2DM, whereas multiple significant associations were reported with type 1DM, including BMI, SBP, and HbA1c [29]. A similar significant moderate association (*r* = 0.54) was reported in the previous and current study between CAN and SBP among type 2DM [29]. Another study looking at the correlates of risk factors with CAN in pre-diabetic and DM people reported SBP as the only factor significantly associated with CAN [30]. In contrast to previous studies, a study in newly diagnosed type 2DM showed that BMI was independently associated with CAN after adjustment for age, sex, HbA1, pulse pressure, triglyceride-to-HDL cholesterol ratio, kidney function parameters, and antihypertensive treatment [31]. Peripheral neuropathy, prolonged QTc, higher age, and longer disease duration were reported to be associated with CAN in a different study among people with type 2DM [32]. Similar factors were also reported in another study with the addition of HbA1c, DBP, and Lower HDL cholesterol associated with CAN, however, in type 1DM [33]. Overall, it is complicated to understand this variation in the findings from different studies, but multiple reasons may contribute to it, such as different types of DM and different methodologies in the assessment of CAN. Some studies did not utilize the total score of the cardiovascular autonomic reflex tests, instead used percentiles of categories (early CAN, confirmed CAN, severe CAN) [31] and some utilized two tests only out of the five tests [33]. Therefore, it is difficult to relate the findings of the current study to the former ones.

In parallel with many previous studies, the current study reported a moderate association between high SBP and CAN. This association between SBP and CAN may be explained simply due to the role of sympathetic activity dominance over the parasympathetic activity in the increase of blood pressure [34, 35]. The function of ANS mainly involves both arms of the ANS working in balance with each other. However, with CAN, the balance between the two arms is absent or reduced. This mainly is manifested by reduced parasympathetic activity and dominant sympathetic influence over the heart and vascular system, especially in the muscular structure [36]. Indeed, sympathetic hyperactivity and higher sympathetic neural discharge are greater among people with type 2DM [37]. Furthermore, in vitro studies, it was found that replication of vascular smooth muscles is increased with catecholamines (e.g., adrenalin, noradrenalin), which may lead to vascular wall hyperplasia [38] and may eventually lead to arterial stiffening [39]. The positive association between SBP and CAN emphasize the importance of controlling blood pressure among people with type 2DM.

Surprisingly, no association was found in the current study between multiple traditional and serological CVD risk factors with Ewing’s score. Among the traditional CVD risk factors, BMI was one of the reported factors associated with CAN. In the current study, the absence of association between BMI or body fats percentage with CAN is perhaps due to the smaller sample size from other studies, which reported an inverse association [29, 31, 40]. This also applies to other CVD risk factors such as age and body fats percentage. The majority of the participants were on various medications, which can have a positive influence on many risk factors studied in the current study. For example, almost half of the participants used statins; thus, serological CVD risk factors such as cholesterol, triglycerides, and HDL were within the normal ranges (see Table 2). This might contribute to the absence of association between the factors mentioned earlier with CAN. Insulin resistance measured via HOMA was also found to be associated with CAN pre-diabetic people [41]. However, in the current study, the smaller sample size and different studied population may have contributed to the lack of detection of this association. These are just speculations, and further studies are needed to clarify these associations with the control of medications when possible.

To the best of knowledge, this is the first study that investigated the association in a cross-sectional study between CRF and CAN in people with type 2DM. Only one study was found assessing this relationship among recently diagnosed people with type 1 and 2 DM. Despite the reduction in CRF in type 2DM people more than type 1DM people, the inverse association between CRF and CAN was reported among people with type 1DM only but not among people with type 2DM [22]. In contrast, the current study found an inverse association between CRF and CAN in people with type 2DM. It is difficult to compare the findings of the current study with the former one due to the variation in methodology in the assessment of ANS function as the current study utilized cardiovascular autonomic reflex tests, whereas the former used heart rate variability (HRV) indices.

To explain the relationship between CRF and CAN, it is well-known that regular exercise and physical activity increase parasympathetic activity and reduce sympathetic hyperactivity in people with type 2DM [42]. Thus, concerning the inverse relationship between CRF and CAN, it is plausible that the reduction of CRF will alter parasympathetic activity via more influence from sympathetic hyperactivity [43]. Improved CRF is known to be associated with better baroreflex sensitivity, which is known to have a better regulatory functioning from ANS, and thus better regulatory function over the heart and vascular system. Regular aerobic exercises may aid ANS adaptation toward parasympathetic predominance via better vagal modulation and reduced sympathetic activity [44]. The association between CRF and CAN reported in the current study indicates the significance of targeting people with type 2DM to be enrolled in exercise training programs to prevent autonomic deficits.

The study has some limitations. Causality between the variables is not possible due to the cross-sectional design of the study. The number of people in this study is relatively small, making some of the commonly reported risk factors in the literature not associated with CAN in the univariate analysis. For ethical reasons, the use of medications was not discontinued or altered. As reported in the study, participants were on various medications that may influence the study’s findings. For example, metformin has shown improved ANS balance in type 2DM [43]; thus, the current study’s results need to be interpreted with caution. Furthermore, due to the variety of medications used by the participants, it was difficult to investigate these medications’ influence. Future studies should include a larger sample and control medications when possible.

## Conclusion

The study results showed a moderate positive correlation between resting SBP and CAN and a moderate negative correlation between VO_2peak_ and CAN in people with type 2DM. Both factors were independent predictors of CAN in people with type 2DM. This may indicate that control of SBP and improvement of CRF has a major contribution to preventing or reducing CAN in people with type 2DM. Future cross-sectional longitudinal studies should investigate if the management of these two factors reduced CAN among these people.

## Data Availability

The data analyzed for this study is available from the author upon reasonable request.

## Abbreviations

ANS: Autonomic nervous system
ACE: Angiotensin-converting enzyme
BMI: Body mass index
BP: Blood pressure
Ca: Calcium
CAN: Cardiovascular autonomic neuropathy
CRF: Cardiorespiratory fitness
CVD: Cardiovascular disease
COPD: Chronic obstructive pulmonary disease
DM: Diabetes mellitus
ECG: Electrocardiograph
ETT: Exercise tolerance test
HDL: High-density lipoprotein
HR: Heart rate
HOMA: Homeostasis model assessment
LDL: Low-density lipoprotein
NSAIDs: Non-steroidal anti-inflammatory drugs
SPSS: Statistical Package for Social Sciences
